# A novel high-throughput molecular counting method with single base-pair resolution enables accurate single-gene NIPT

**DOI:** 10.1038/s41598-019-50378-8

**Published:** 2019-10-07

**Authors:** David S. Tsao, Sukrit Silas, Brian P. Landry, Nelda P. Itzep, Amy B. Nguyen, Samuel Greenberg, Celeste K. Kanne, Vivien A. Sheehan, Rani Sharma, Rahul Shukla, Prem N. Arora, Oguzhan Atay

**Affiliations:** 1 BillionToOne Inc., Menlo Park, CA 94025 USA; 2 Department of Cellular and Molecular Pharmacology, University of California, San Francisco, San Francisco, CA 94158 USA; 3 Department of Pediatrics, Division of Hematology/Oncology, Baylor College of Medicine, Houston, TX 77030 United States; 4 SeqIndia Labs Pvt. Ltd., New Delhi, India; 5 Yashoda Super Speciality Hospitals, H-1 Kaushambi, Ghaziabad, Uttar Pradesh 201001 India

**Keywords:** Assay systems, Assay systems, DNA sequencing, DNA sequencing

## Abstract

Next-generation DNA sequencing is currently limited by an inability to accurately count the number of input DNA molecules. Molecular counting is particularly needed when accurate quantification is required for diagnostic purposes, such as in single gene non-invasive prenatal testing (sgNIPT) and liquid biopsy. We developed Quantitative Counting Template (QCT) molecular counting to reconstruct the number of input DNA molecules using sequencing data. We then used QCT molecular counting to develop sgNIPTs of sickle cell disease, cystic fibrosis, spinal muscular atrophy, alpha-thalassemia, and beta-thalassemia. The analytical sensitivity and specificity of sgNIPT was >98% and >99%, respectively. Validation of sgNIPTs was further performed with maternal blood samples collected during pregnancy, and sgNIPTs were 100% concordant with newborn follow-up.

## Introduction

Next-generation sequencing (NGS) has revolutionized both research and clinical practice. In 2001, a global effort was required to sequence the first human genome. Since then, there has been extraordinary progress in DNA sequencing technology, and genome sequencing is now routinely performed by small research and clinical groups^1^. In research settings, significant scientific advances have been made through innovative experimental designs that generate high throughput data resolvable by NGS^2,3^. In the clinic, performing whole exome sequencing is now considered the standard of care when congenital and neurodevelopmental disorders cannot otherwise be diagnosed^4^.

Clinical applications of genome sequencing technology to the clinic have been even more widely adopted in the fields of prenatal care and oncology. Targeted oncologic therapies are now indicated when specific genetic profiles are found in tumor DNA. Moreover, DNA sequencing of biopsied tissue is now an FDA-approved procedure that is covered by health insurance policies^5,6^. It has also been shown that circulating tumor DNA can be found in the cell-free DNA (cfDNA) purified from plasma^7^. This has led to ‘liquid biopsies’ that detect cancer mutations by DNA sequencing of a non-invasive blood sample^8^. In prenatal care, cfDNA of fetal origin obtained from maternal blood is used to detect fetal aneuploidies as early as the 10th week of gestation^9,10^. These non-invasive prenatal tests (NIPT) are routinely used in clinical care and are also covered by health insurance^11^.

To date, cfDNA-based tests have mostly been limited to detection of chromosomal abnormalities or large structural variants in prenatal testing^10^ and screening for abnormal sequences in late-stage cancers in liquid biopsy^12^. Yet, several prevalent genetic disorders are caused by single nucleotide variants (SNV) or a single gene copy number change. In liquid biopsy, even though copy number variation is a hallmark of cancer^13^, current FDA-approved diagnostics are limited to resolving a 30% to 120% increase in the copy number found in cfDNA and therefore are only applicable to late-stage cancers^14,15^. NIPTs are especially needed to improve diagnosis of sickle cell disease, alpha-thalassemia, beta-thalassemia, cystic fibrosis, and spinal muscular atrophy. Medical guidelines recommend that all pregnancies should be screened for these disorders^16,17^. Hemoglobinopathies in particular, i.e., sickle cell disease, beta-thalassemia, and alpha-thalassemia, are the most common genetic disorders in the world, affecting more than 300,000 births each year^18^. In the US, 1 in 12 African-Americans and 1.5% of all newborns are carriers for sickle cell disease^19^. In addition, the carrier rates for cystic fibrosis and spinal muscular atrophy are 3% and 2%, respectively^20,21^. NIPTs to screen for these recessively inherited single gene disorders are currently not available. Because of this, the only way to diagnose these disorders in the fetus is through invasive methods such as amniocentesis or chorionic villus sampling (CVS). These procedures carry a small risk of pregnancy loss^22,23^.

Prenatal diagnostic tests for recessively inherited disorders are particularly challenging because they require precise DNA quantification over a large background level. Traditional library preparation methods for NGS can introduce significant biases that obscure the relationship between the number of input DNA molecules and final sequencing output. Without the ability to perform absolute quantification, NGS cannot reliably quantify fetal SNVs in prenatal testing, detect gene amplifications that are present in Stage 1–2 cancers, or sensitively detect rare cell-free tumor DNA sequences that may only be present at 1–10 molecules in the sample^24^. Of note, because the major sources of noise are introduced during amplification and library preparation, increased sequencing depth does not necessarily result in improved accuracy^25^. Currently, the only method for absolute quantification of DNA molecules is digital PCR (dPCR), but its limited multiplexability hinders its applicability to single-gene NIPT or liquid biopsies^26,27^.

**Figure 1:**
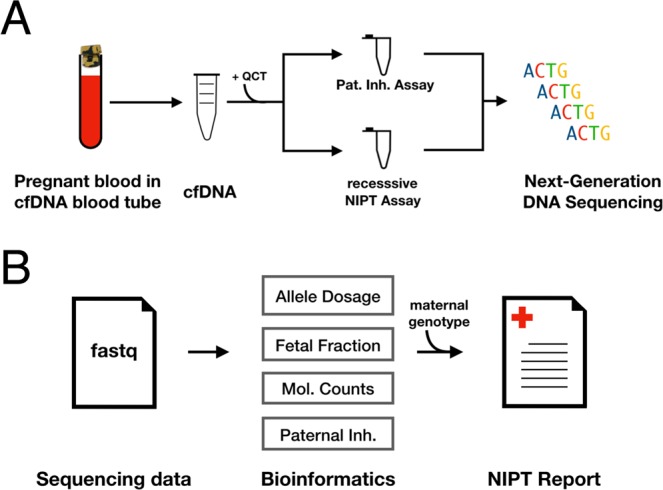
Overview of single-gene NIPT. (**A**) Clinical workflow uses a single tube of maternal blood. Amplicon-based assays are performed and sequenced using an Illumina Miseq instrument. (**B**) Bioinformatic analyses recover the dosage of pathogenic alleles, the fraction of cfDNA isolated from maternal blood that is of fetal origin, the number of DNA molecules assayed, and paternal inheritance of variants not found in the mother’s genotype. These analyses are combined with the maternal genotype to perform a statistical analysis of fetal genotype, resulting in an NIPT report.

In this study we developed a sequencing-based method for counting molecules with single base-pair resolution using Quantitative Counting Template (QCT) molecules. Because molecular abundance information in sequencing read depth data is typically corrupted by library preparation, QCTs encode molecular abundances prior to PCR amplification and subsequent library preparation. Absolute quantification of DNA molecules was then decoded via customized bioinformatic analyses. We then applied QCT molecular counting to NIPTs for Mendelian disorders, including sickle cell disease, thalassemias, cystic fibrosis, and spinal muscular atrophy. These NIPTs require only a single 10 mL sample of maternal blood (Fig. 1). NIPTs were validated with preclinical samples (estimated sensitivity of >98% and specificity of >99%) comprised of genomic DNA sheared to mimic the fragmentation of cfDNA. Crucially, reliable NIPT results were only obtainable via accurate molecular counting of these genes. We also performed NIPT assays on non-pregnant cell-free DNA control samples to show the concordance of sheared DNA samples with cfDNA. Finally, we performed NIPT assays on maternal blood samples obtained from pregnant women. All NIPT results were 100% concordant with the newborn genotype, even in challenging samples with fetal fractions as low as 5%.

## Results

### High throughput molecular counting with single base pair resolution

**Figure 2:**
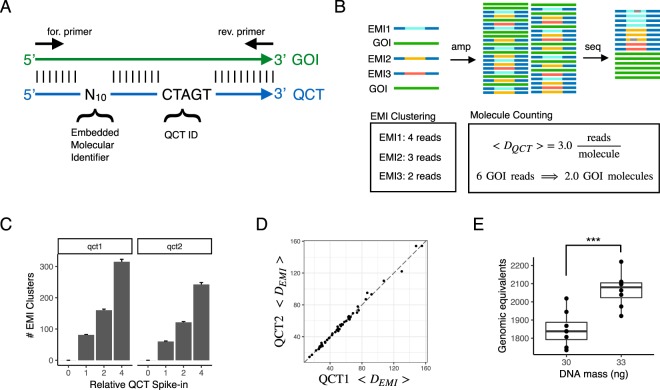
Absolute quantification of genomic equivalents by QCT molecules. (**A**) Sequence design of QCT molecule pools. QCTs are designed to co-amplify with a gene-of-interest (GOI). An Embedded Molecular Identifier comprised of randomized bases ensures each QCT molecule has a unique sequence. (**B**) Schematic of QCT molecular counting workflow. QCT molecules are added to the DNA sample, and the mixture is amplified and sequenced. Clustering analysis identifies and corrects for any amplification or sequencing errors (gray bars). The number of GOI molecules in the sample is calculated from the GOI read depth and the average number of reads per QCT molecule. (**C**) Two QCT pools, QCT1 and QCT2, were synthesized with different QCT IDs and diluted to approximately 100 molecules at 1x dilution. The barplot shows the mean across 24 PCR replicates at each dilution factor. Error bar is 1 standard error of the mean. (**D**) The average read depth per QCT1 and QCT2 in a PCR tube is shown for the 48 PCR replicates at dilution factors 1x and 2x. Dashed line has slope = 1, intercept = 0. (**E**) QCT1 DNA was added to either 30 ng or 33 ng of sheared genomic DNA and processed using the workflow in panel B. About 100 EMI clusters were found per PCR. At 30 ng, the assayable sheared DNA mean was 1849 molecules (CV = 5.2%, n = 8); at 33 ng the mean was 2066 molecules (CV = 4.4%, n = 8). The mean GE measured for 33 ng was significantly greater that that for 30 ng (*p* = 0.00019, one sided t-test).

We first developed a technique to count the number of DNA molecules in a PCR using amplicon NGS workflows (Fig. 2). In this assay, a number of synthesized DNA molecules (Quantitative Counting Templates, QCTs) are spiked into the cell-free DNA specimen prior to PCR amplification. A given QCT sequence is designed to co-amplify at the same rate as its corresponding gene-of-interest by incorporating homologous regions, especially in PCR priming sites (Fig. 2A). QCTs are designed such that the number of molecules added can be independently calculated from sequencing data. The relationship between read depth and molecular counts is then used to determine the gene-of-interest molecular count in the input sample. To facilitate counting of the number of QCT molecules spiked into each tube, the QCT sequence contains a barcode comprised of 10 randomized bases in which A, C, T, or G is stochastically incorporated during oligo synthesis. Because up to 4^10 ^ QCT sequences are synthesized in a pool and only ~100 to 1000 QCT molecules are spiked into a PCR amplification, it is exceedingly unlikely for any two QCT molecules to have the same sequence. The randomized sequence of each molecule thus comprises an Embedded Molecular Index (EMI) that identifies the molecule and its amplification progeny (Fig. 2B). After PCR amplification and DNA sequencing, the number of QCT molecules added to each specimen should correspond to the number of EMI sequence clusters observed, and the gene-of-interest molecular count is determined.

To test our ability to count the number of QCT molecules present in a reaction, we added 4x, 2x, 1x, or 0x QCT molecules to PCRs, with 1x corresponding to approximately 100 QCT molecules. Twenty-four replicates were assayed at each spike-in level, for a total of 96 reactions. EMI sequences from the same PCR tube that differ by up to 2 mismatches were clustered together in order to avoid counting spurious indexes that arise due to sequencing error. We observed the expected relationship between EMI sequence clusters and the $\frac{1}{2}$ dilution series of QCT molecules (Fig. 2C). The number of QCT molecules added at each level is expected to vary among replicates due to sampling noise; addition of *N* molecules to a reaction should exhibit a standard deviation $\sqrt{N}$ according to Poisson statistics. This effect was observed in our data, as the variance of QCT molecule counts increased with more molecules added (Fig. 2C).

We confirmed that the EMI cluster numbers were robust to read-depth variation by repeating cluster analysis on sequencing data that had been subsampled to $\frac{1}{2}$ of its original read depth (Fig. S1A). The number of EMI clusters obtained from subsampled data matched perfectly with the full sequencing data, except when there were fewer than 10 reads per EMI cluster. This result suggests that a sequencing depth of 10 reads per molecule is sufficient to generate robust results.

Quantification of assayable human genomic equivalents (number of haploid genome copies in the sample) relies on the assumption that the gene-of-interest and QCT DNA co-amplify at exactly the same rate in PCR. Therefore, QCT sequences are designed to use the same PCR primer binding sites and to generate the same length amplicon as the gene. However, because the internal sequence differs between the QCT and the corresponding gene, QCT molecules could hypothetically be amplified at a different rate than that of the gene. To ensure that the PCR amplification is robust to sequence composition, we examined the average depth per molecule, $\langle {D}_{QCT}\rangle$, of two QCT pools. Pools QCT1 and QCT2 were synthesized with QCT IDs TCGCC and CTAGT, respectively. Approximately 100–200 molecules of each QCT pool were added per PCR, and the average read depth per molecule from each pool was compared across the 48 PCRs at 1x and 2x dilutions (Fig. 2D). Because QCT pools incorporate 10 randomized bases in addition to the 5 mismatches in the QCT IDs, the sequences of the ~100 QCT molecules from each pool in a PCR assay are highly diverse. In each PCR assay, $\langle {D}_{QCT1}\rangle$ and $\langle {D}_{QCT2}\rangle$ were highly consistent. Even when PCR amplification biases resulted in an 8-fold difference in the sequencing read-depth, $\langle {D}_{QCT2}\rangle$ was highly correlated with $\langle {D}_{QCT1}\rangle$ with *R*^2^ > 0.99 (Fig. 2D), and the mean difference between $\langle {D}_{QCT1}\rangle$ and $\langle {D}_{QCT2}\rangle$ was only 0.006% (Fig. S1B). This suggests that the QCT method completely corrects for amplification differences in PCR. Moreover, because the sequence differences between the two QCT pools was much higher than the sequence differences between a a typical QCT and its corresponding gene, we expect ~0.006% to be an upper bound on the amplification difference between QCTs and the respective genes of interest. Introducing distinct sets of QCT pools into all reactions also provides an internally controlled upper bound for the error of $\langle {D}_{QCT}\rangle$ measurements, which can be important for detecting assay degradation on a per-sample basis due to low sample quality or interference by contaminants (e.g., ethanol or salt carryover from DNA extraction).

We then sought to quantify the amount of human DNA present in a sample using QCTs. The number of DNA molecules corresponding to the gene-of-interest was calculated as $GE=\frac{{D}_{GOI}}{\langle {D}_{QCT}\rangle }$; where *GE* is the haploid genome equivalents, and *D*_*GOI*_ is the read depth of the gene-of-interest (Fig. 2B). To more closely mimic the fragmentation pattern of cfDNA, human DNA was acoustically sheared to a mean fragment length of ~150 bp. A dilution series of 2–36 ng sheared DNA was prepared with approximately 200 QCT molecules per reaction (Fig. S1C). PCR primers targeting a 150 bp region of *HBB* exon 1 were used to co-amplify the QCT and sheared DNA molecules to yield a 150 bp amplicon. Because not all sheared DNA molecules span both primer binding sequences, only a fraction of the sheared DNA would be amplifiable by PCR. After we counted the number of amplifiable sheared DNA molecules, we performed a regression analysis to show that 64 GE/ng of sheared DNA was detectable, compared to the theoretical maximum of 278 GE/ng corresponding to a haploid genome mass of 3.6 pg (Fig. S1C). This finding demonstrates the importance of directly measuring assayable genomic equivalents as opposed to DNA mass alone, because cfDNA is also fragmented with a mean length of ~167 bp^28^. We then established the precision of GE estimates by measuring a smaller range of 30–33 ng of sheared DNA with QCT molecules. Again, we reproducibly obtained genomic equivalents of ~60 GE/ng of sheared DNA (Fig. 2E). Measured GE increased linearly with more DNA mass, increasing from 1850 GE at 30 ng of DNA to 2070 GE at 33 ng of DNA. The coefficient of variation (CV) of *HBB* molecular counts was 4.8%. Because the overall CV is affected by pipetting precision and Poisson sampling of limited DNA molecules (2.2% CV at 2000 molecules), 4.8% CV is the upper bound for QCT molecular counting precision.

### Ultra-rare variant calls enabled by QCTs

**Figure 3:**
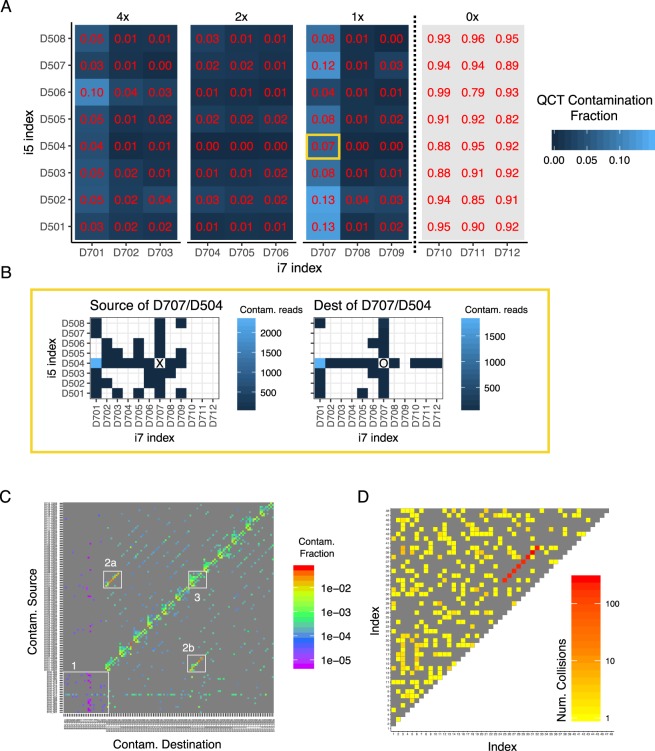
QCT tracking analysis quantifies contamination and sample mixups. (**A**) The experiment from Fig. 2C was analyzed for contamination using QCTs. Either 4x, 2x, 1x or 0 QCT molecules were added to 96 PCRs and sequenced on an Illumina Miseq lane using Truseq-style index pairs. Each EMI cluster for a given i5/i7 index pair was classified as contamination if (i) the read depth was below threshold (see Methods and Fig. S2), and (ii) the same EMI cluster was found to originate from another index pair. The QCT contamination fraction for a given index pair is the ratio of contaminating reads over total reads. (**B**) Demonstration of QCT tracking analysis to identify the source of contamination. Contaminating EMI clusters in the D707/D504 index pair (yellow box, **A**) are found to mostly originate from D701/D504 (left), and likewise, contaminants in D707/D504 are found to originate from D707/D504 (right). (**C**) QCT contamination tracking shows dual-unique indexes drastically reduce index misassignment. Both dual-unique indexes and Truseq-style index pairs were used on 120 samples prepared and sequenced in batch. Contamination source/destination is shown for all pairs of the 120 PCRs. The reactions that used dual-unique indexes (inset 1) typically had 0.006% contamination compared to 0.5% contamination using Truseq-style index pairs, particularly for D701 and D704 (insets 2a and 2b). Relatively high contamination was also observed in Truseq-style PCRs that had a D7xx index in common (example in inset 3), which is consistent with index misassignment. (**D**) Pairwise analysis of QCT fingerprints identifies sample mixups. Forty-eight PCRs were processed in parallel, dual unique indexed, and sequenced on a Miseq lane. The similarity of QCT fingerprints is quantified as the number of high read depth EMI clusters in common (i.e. collisions) between two reactions. The number of collisions for all pairs of PCRs is shown.

A particular challenge in detecting rare variants is false-positives due to contamination from another positive sample. Contamination can be particularly problematic when multiple libraries are processed in parallel and sequenced in the same run^29,30^. Because the sequence diversity of QCT molecules aliquoted into each reaction is very small compared to the total QCT diversity in each synthesized pool, we realized that the set of EMI cluster sequences associated with each PCR can be used as a fingerprint to identify the sample. Given a total pool diversity of 1 million sequences, ~10^443^ fingerprints are possible if 100 sequences are added to each PCR. Analysis of PCR fingerprints across the entire sequencing workflow can then be used to rule out the possibility that detected rare variants in any given reaction are from sample cross-contamination (Fig. 3).

We further analyzed the experiment performed in Fig. 2C to quantify contamination using QCT fingerprints. The sequencing depth for each EMI cluster in a PCR library was distinctly bimodal, with most EMI sequence clusters read at depth >30x. A minority of EMI sequencing reads were also present at depth 1–2x (Fig. S2). EMI sequencing clusters were classified as high-depth or low-depth (see Methods). Low-depth EMI sequences could arise from (i) sequencing error, (ii) errors introduced during PCR amplification, (iii) cross-contamination during sample handling, or (iv) index misassignment^29^. A low-depth EMI sequence cluster (typically 1–2x) observed in a PCR was classified as a contaminant if it was also observed at high-depth in a different PCR well. We then computed the contamination fraction for each PCR as the number of contaminated QCT reads over total QCT reads (Fig. 3A). The PCRs with 0x QCT molecules should therefore register 100% contamination. QCT contamination analysis measured >90% contamination in 0x QCT wells, suggesting that this method is highly sensitive for detecting contamination. The remaining ~10% of undetected contamination could be due to sequencing error or contamination that had occurred prior to PCR amplification. Unexpectedly, we found that PCR libraries barcoded with D701 and D707 indexes had high levels of total contamination consistently >5% and as high as 13% (Fig. 3A). A more granular analysis of contamination that traced contamination sources on a per-tube level revealed that for the reaction indexed by D707/D504, nearly all of the contamination originated from the D701/D504 reaction. Conversely, the D701/D504 well was the main destination of contaminating EMIs that originated from the D707/D504 reaction (Fig. 3B). Similar cross-contamination patterns were observed in the other wells indexed by D701 and D707. These data suggested to us that our D701 and D707 indexes themselves had become cross-contaminated, perhaps during oligo synthesis or index preparation. We also observed that most of the remaining contamination occurred in wells that have a D7xx or D5xx index in common, which is consistent with contamination due to index misassignment. To address these issues, we prepared and sequenced a new library in which barcoding was performed with either dual unique indexes^31^ or the previously used D5xx and D7xx index pairs. Tru-seq HT style combinatorial indexing using D7xx/D5xx pairs again resulted in striking levels of contamination, with a median contamination of 0.5% (maximum 8.9%). Dual unique indexes reduced the observed contamination to 0.006% (maximum 0.03%), a nearly 100-fold improvement (Figs 3C and S3).

In addition to contamination quantification, QCT analysis can also detect sample mixups due to operator error during library preparation barcoding. If the same QCT fingerprint is observed in multiple samples at high read depth, this would indicate that a single PCR was indexed to multiple barcodes. To quantify this, a sample collision score is defined as the number of high read-depth EMI clusters in common between two PCRs. We measured the number of colliding EMI sequences across all pairs of PCRs in an experiment where 8 reactions were barcoded twice in the same sequencing run. When the pairwise collisions are plotted, barcodes 25–32 immediately stand out because they share ~150 EMI clusters in common with corresponding barcodes 33–40. This approach can therefore be used to identify common operator errors that would result in a reduced number of fingerprints in the sequencing data (Fig. 3D).

### Single-gene NIPT enabled by single base pair molecular counting

**Figure 4:**
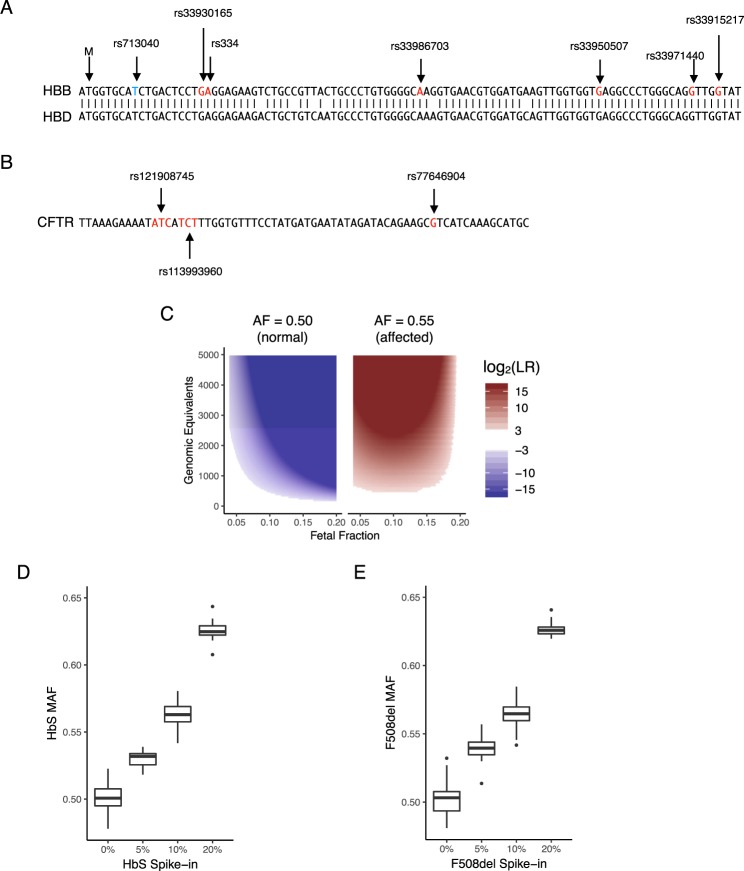
NIPT for sickle cell disease and cystic fibrosis by amplicon NGS. (**A**) Target region of *HBB* exon 1 NIPT and alignment with *HBD*. Start codon is shown as ‘M’. rs713040 is a benign variant with high allele frequency. rs33930165 and rs334 are pathogenic variants. (**B**) Target region for CFTR NIPT of the F508del variant. (**C**) The NIPT assay uses pathogenic allele fraction, fetal fraction, and assayed genomic equivalents data to compute a likelihood ratio (LR) for an affected homozygous vs heterozygous fetus. Regions where $\frac{1}{8} < {\rm{LR}} < 8$ are shaded white to represent no-call regions. Affected NIPT calls are shaded blue, and non-affected calls are shaded red. (**D**) Validation of allele fraction measurement for sickle cell disease. Genomic DNA corresponding to a heterozygous sickle cell trait (NA20838) mother was sheared to 150 bp and 0 to 10% sheared sickle cell disease DNA (NA16265) was mixed in. The HbS allele fraction (AF) was measured by amplicon NGS sequencing. When no SCD DNA is mixed in, the mean HbS AF = 0.501 (n = 80, CV = 1.8%). With 10% SCD DNA, mean HbS AF = 0.563 (n = 80, cv = 1.6%). QCT analysis measured 2200 GE in these assays. The total number of samples shown is n = 184. (**E**) Validation of allele fraction measurement for cystic fibrosis. Sheared homozygous cystic fibrosis variant F508del was spiked into 30 ng heterozygous F508del sheared DNA at the indicated proportions. F508del MAF was calculated with the following formula $\frac{{D}_{F508}}{{D}_{wt}+{D}_{F508}}s$, where *s* is a shearing correction factor. The shearing correction factor compensates for the increased presence of the 3 bp shorter F508del allele present in sonicated DNA. The correction factor is derived from a geometric distribution of sheared fragments with a mean length of 150 using the following formula $s={(1-\frac{1}{150})}^{100}/{(1-\frac{1}{150})}^{97}=0.980$.

We next designed PCR assays for amplifying regions responsible for the most common genotypes of sickle cell disease, cystic fibrosis, spinal muscular atrophy (SMA), beta-thalassemia, and alpha-thalassemia (Figs 4 and S4–S7). Sickle cell disease is most commonly caused by the rs334 (HbS) and rs33930165 (HbC) variants within exon 1 of *HBB*; over 90% of sickle cell disease cases are either HbSS or HbSC^32^. Approximately 1 in 12 African-Americans^19^ are sickle cell trait carriers (HbAS). Because the paralogous genes *HBB* and *HBD* are highly similar in this region, >2 bp of variation between *HBB* and *HBD* was included in the targeted region to ensure accurate read mapping (Fig. 4A). Similarly, cystic fibrosis is caused by recessive inheritance of mutations in *CFTR* (Fig. 4B). Over 23 pathogenic *CFTR* variants are commonly screened for^33^. Of these, the *CFTR* F508del variant is the most common, with over 70% of cystic fibrosis patients carrying one or more F508del variant^34^. SMA and alpha-thalassemia are typically caused by gene deletions, which requires modified designs to quantify the pathogenic allele fraction (Figs S6 and S7).

 Single-gene NIPT analysis combines the pathogenic allele fraction (AF) observed from maternal blood, the number of input DNA molecules that were assayed for the allele fraction measurement, and the fetal fraction measurement into a statistical model (Fig. 4C)^35,36^. We first validated that accurate allele fraction measurements could be obtained from *HBB* exon 1. We generated ersatz cfDNA samples by shearing genomic sickle cell DNA to ~150 bp. Approximately 30 ng total of sheared DNA was added to each PCR assay, and QCT analysis showed that 2200 GE of *HBB* exon 1 were amplified from sheared DNA. In the ersatz samples of heterozygous HbAS DNA (NA20838, Coriell), we measured a mean HbS allele fraction of 0.501 ± 0.0010 standard error of the mean (SEM) across 80 PCR replicates (Fig. 4D), indicating that *HBB* alleles are amplified without introducing any bias. The CV of these replicates was 1.6–2.2% (95% confidence interval). Importantly, the CV is in accordance with the Poisson noise of 2.1% associated with 2200 molecules, suggesting that the assay for *HBB* allele fraction is operating at the physical limit of counting statistics.

We next prepared a sheared DNA mixture of 90% HbAS and 10% HbSS sickle cell disease DNA (NA16265) to mimic cfDNA from a sickle cell trait carrier pregnancy with 10% sickle cell disease (SCD) fetal fraction. Mixtures were also prepared to correspond to an affected fetus at 5% and 20% SCD fetal fractions. The allele fraction measurement of sheared NA20838 alone (the 0% HbS Spike-in mixture), is representative of a sickle cell carrier pregnancy with an unaffected sickle cell trait fetus. Out of 184 samples, 80 corresponded to a carrier fetus and 104 corresponded to an affected fetus. The large separation between the 0% and 10% HbS spike-in mixtures shows that HbS allele fraction differences for a sickle cell trait (heterozygous) vs sickle cell disease (homozygous) fetus can be easily discriminated (Fig. 4D). The HbS allele fraction was then used to classify affected and unaffected ‘fetal status’ based on a statistical model of likelihood ratio that incorporates molecular count information (see Methods). All samples with >5% HbS spike-in were correctly identified as ‘affected’ for sickle cell disease NIPT (Table S1). Although there is a slight overlap between the 0% and 5% HbS mixtures that led to 4 no-calls, all NIPT calls (n = 184) correctly identified the samples as affected vs. unaffected. Similar results were obtained for >200 samples at mixtures ranging from 0% to 20% for cystic fibrosis, SMA, and alpha-thalassemia (Figs 4E and S4–S7). To gain further insight into the analytical performance of single-gene NIPT, we performed Monte Carlo analysis for sensitivity and specificity of NIPT from maternal blood only (Table S2). Assuming a paternal carrier rate of 1 in 12 and a 10% fetal fraction, the sensitivity and specificity of NIPT analysis were both >99%. Even at 5% fetal fraction, the sensitivity and specificity was >98% and >99%, respectively.

**Figure 5:**
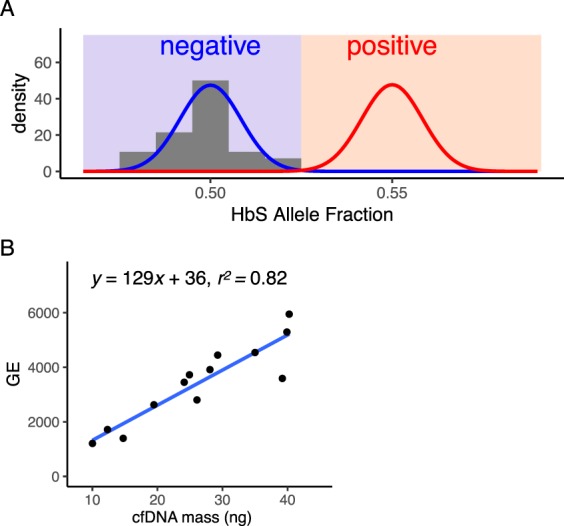
SCD NIPT on negative control cfDNA resulted in no false-positives. Optimized *HBB* probes were validated for use on cfDNA by performing the NIPT assay on 10 mL of venous blood from non-pregnant compound heterozygotes (HbAS or HbSC). The mean HbS AF was 0.498 with a coefficient of variation of 2.2% (n = 30). The mean number of *HBB* molecules assayed in each blood tube was 3500. Our results agree with the expected HbS AF = 1/2 and CV = 1.7%; the histogram of measured HbS AF corresponds very well with the theoretical binomial distribution in blue (AF = 1/2, n = 3500). Assuming that positive cases have a 10% fetal fraction (red curve), none of the negative controls would have been called as positive for fetal SCD. (**B**) Assayed genomic equivalents of *HBB* exon 1 in cfDNA. The concentration of 13 cfDNA samples was quantified by Qubit to determine the mass of cfDNA used in the *HBB* assay. On average, 1 ng of cfDNA resulted in the capture of 129 haploid genomic equivalents of *HBB*.

To confirm that sheared DNA behaves similarly to cell-free DNA, we next obtained 30 blood samples from male and female patients that were compound heterozygotes for sickle cell disease at Baylor College of Medicine (Fig. 5). cfDNA was purified from blood plasma and the *HBB* allele fraction assay was performed. The results of the *HBB* allele fraction assay again agreed with the expected $\frac{1}{2}$ allele fraction (Fig. 5A). On average, 3500 GE of *HBB* were assayed for each 10 mL blood sample. We measured 2.2% CV (1.6–2.9% 95% CI), which was in good agreement with the expected CV = 1.7% associated with Poisson counting of 3500 GE. The proportion of *HBB* DNA amenable to PCR amplification was 129 GE/ng cfDNA (Fig. 5B), which is almost twice what we previously observed for sheared DNA (Fig. 2D). The increased numbers of assayable GE from cfDNA shows that the analytical validation performed with ersatz samples was more challenging than for cfDNA blood samples. This difference in capture efficiency could be because acoustically sheared DNA (~150 bp) was more fragmented than cfDNA (165 bp) and/or differences between acoustic shearing and biological mechanisms of cfDNA fragmentation^28^. Because none of the blood samples were taken from pregnancies, they also serve as negative controls for NIPT analysis. As expected, all of the allele fraction measurements in these non-pregnant controls would have resulted in negative NIPT results (Fig. 5A).

### Clinical validation of single-gene NIPT

We next obtained maternal blood and saliva samples from 208 healthy pregnant donors from Yashoda Hospital, Ghaziabad, India. Ethical clearance was obtained from the Yashoda Institutional Ethics Committee (IEC: ECR/970/Inst/UP/2017). When we genotyped these samples, there were only 3 pregnant donors who were beta-thalassemia carriers. Although beta-thalassemia is common in India with a carrier rate as high as 5%, the distribution of pathogenic alleles is heterogeneous across subpopulations and geographic areas^37^. Therefore, to clinically validate *HBB* NIPT by molecular counting, we performed NIPT analysis for a linked, benign variant that is located within the target region of *HBB* exon 1 (Fig. 4A). Because *HBB* NIPT is an amplicon-based NGS assay, the ability to detect fetal inheritance of this variant (rs713040) is identical to that of SCD or any other pathogenic variants that are found in the target region of exon 1 (e.g., HbS; HbC; HbE; IVS1,1; IVS1,5). Moreover, because this variant has an extremely high minor allele frequency (0.2), we were able to obtain all maternal-fetal genotype combinations that represent healthy and disease states.

Follow-up *HBB* genotyping of newborns was obtained for 52 pregnancies. However, the amount of cfDNA recovered from corresponding maternal plasma samples using the *HBB* allele fraction assay was found to be abnormally low (mean GE = 996, min GE = 21) compared to the cfDNA purified from negative controls collected at Baylor College of Medicine (mean GE = 3500, min GE = 1200; Fig. 5). Fifteen of the pregnant cfDNA samples contained <200 GE of assayed *HBB*. Because 200 GE corresponds to <2 ng of cfDNA, these 15 samples were excluded from further analysis. Previous reports have shown that ~4000 GE should be recoverable from a 10 mL blood sample^35,38^. The paucity of cfDNA recovered from these samples may most readily be explained by DNA degradation during sub-optimal extended storage at −20 ^o^C for 5 to 8 months between sample collection and sequencing.

Our approach for NIPT integrates measurements of (i) fraction of fetal DNA present in cfDNA, (ii) molecular counts of assayed cfDNA, (iii) allele fraction of the maternal variant, and (iv) allele fraction of any variants that are not present in the maternal genotype (distinct paternally inherited variants). Approximately 1/4 of each purified cfDNA sample was used to determine the fetal fraction using a custom amplicon NGS assay that interrogates 86 common SNVs across all autosomal chromosomes (Fig. S8). QCT molecules were added to the remaining cfDNA and the *HBB* allele fraction assay was performed to determine molecular counts and allele fractions of *HBB* exon 1 variants. These measurements were then used in a statistical model to determine the genotype of the fetus. Because a fetus inherits one allele from each parent, in cases where the maternal genotype is homozygous, the detection of any non-maternal allele indicates that the fetus is heterozygous for that variant, i.e., inherited a paternal allele (paternal inheritance). On the other hand, for a sample where the maternal genotype is heterozygous, NIPT for recessive inheritance requires precise quantification of allele fraction to determine whether fetal alleles are contributing to an observed allele fraction significantly different from $\frac{1}{2}$. For example, the expected variant allele fraction (VAF) in cfDNA for a heterozygous fetus will remain at $\frac{1}{2}$, the same as the maternal level. However, if the fetus is homozygous for the variant, i.e., the fetus has inherited two identical alleles with the pathogenic variant, the VAF from the cfDNA sample should increase to 0.55 for a sample with 10% fetal fraction, to 0.60 VAF for 20% fetal fraction, and so on (recessive inheritance). The probability distributions of these allele fractions depends on the molecular counts due to Poisson counting noise (Fig. 4C).

**Figure 6:**
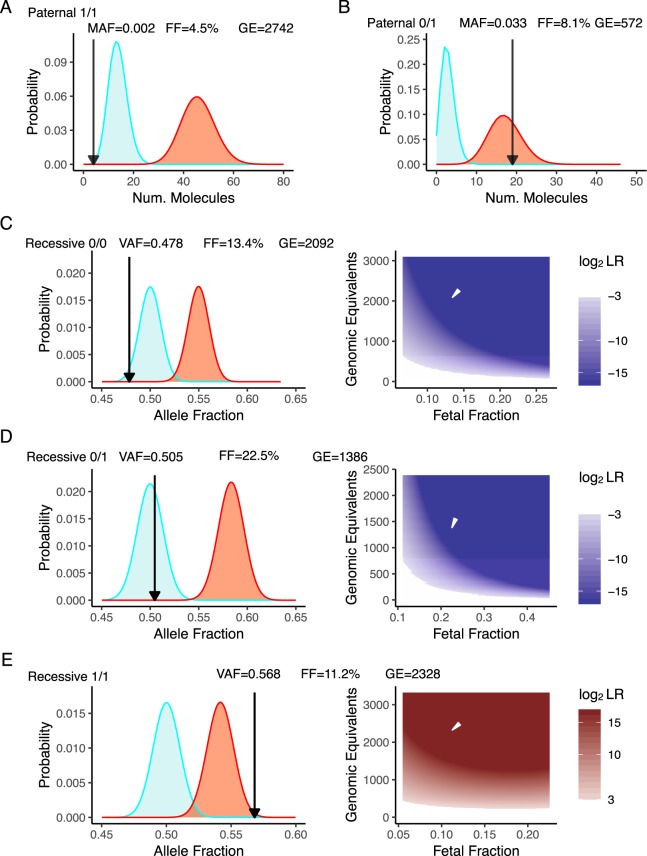
NIPT of rs713040 in *HBB* exon 1. (**A**) Probability distribution of sample 04B for paternal inheritance resulting in a NIPT homozygous call. (**B**) Probability distribution of sample 38 C for paternal inheritance resulting in a NIPT heterozygous call. Black and white arrows indicate diagnostic measurements of allele fraction, GE, and fetal fraction. (**C**) Negative call for recessive NIPT resulting in a homozygous child. (**D**) Negative recessive NIPT call resulting in a heterozygous child. (**E**) Positive recessive NIPT call resulting in an ‘affected’ child.

In samples that had a homozygous (C/C reference or T/T variant) maternal genotype for the benign variant rs713040, we performed *HBB* NIPT by detecting a distinct paternally inherited fetal allele (n = 14; Table 1). In these pregnancies, a heterozygous fetal genotype can occur only when the fetus inherits a paternal allele different from the maternal allele, and the resultant minor allele fraction (MAF) in cfDNA is expected to be $\frac{1}{2}$ of the fetal fraction (e.g. MAF of 0.05 when fetal fraction is 10%). Likewise, a homozygous fetus matching the genotype of the mother is determined by the absence of a paternal allele. This detection requires distinguishing a true, rare variant, i.e., the paternal allele, from any other sources of low-frequency variants introduced either by contamination, index misassignment, amplification, and/or sequencing errors. For these samples, QCT analysis indicated that sample contamination and index misassignment was negligible (median = 0.01%, max = 0.2%). Then, we used a statistical model to ensure that any observed minor alleles could not be explained by sequencing/polymerase error (Fig. 6A,B; discussed below). Because QCT analysis showed that these samples were sequenced to >50 reads/molecule, the contribution of read depth to noise is negligible, and sampling noise is nearly entirely due to the number of DNA molecules assayed. To rule out the possibility that the observed minor allele molecules are present due to sequencing error, we calculated two probability distributions for each sample: one for background sequencing error and the second for a paternally inherited allele. The fetal genotype is then determined by which probability distribution more closely matches the observed result. Hypothetically, the measured allele fraction could occur within a range where the two probability distributions overlap significantly and result in an ambiguous fetal genotype. The likelihood ratio (LR) roughly corresponds to how likely it is for the observed measurement to be positive or negative, with LR > 1 indicating increased evidence for a positive result (affected fetus). To avoid calling NIPT results on inconclusive evidence, a no-call threshold was set at $\frac{1}{8} < LR < 8$, a standard that has previously been used in single-gene NIPT^35^. Examples of this analysis are shown in Fig. 6A,B for the cases of a homozygous and heterozygous fetus, respectively. The LR for all 14 paternal inheritance cases exceeded 10,000-fold, and *HBB* genotyping of newborns resulting from these pregnancies confirmed that all of the NIPT calls were correct. This was true even in highly challenging samples with limited fetal fraction (<5%) or quantity of cfDNA available (<250 GE). Out of the 14 maternal blood samples analyzed for paternal inheritance, 2 were homozygous negative C/C, 2 were heterozygous negative C/T, and 10 were homozygous positive T/T (Table 1).

**Table 1:** NIPT for paternal inheritance of rs713040.

Sample	Fetal Fraction	VAF	Num. Mol.	LR	NIPT	Neonate GT
35A	0.088	0.004	1159	1.5e + 09	0/0	0/0
07B	0.272	0.009	482	5.9e + 15	0/0	0/0
38C	0.081	0.033	572	2.1e − 09	0/1	0/1
49E	0.174	0.961	436	2.0e − 08	0/1	0/1
38B	0.228	0.998	871	4.3e + 27	1/1	1/1
52E	0.128	0.998	248	4.7e + 04	1/1	1/1
17D	0.172	0.998	585	1.6e + 13	1/1	1/1
18C	0.123	0.998	636	3.6e + 09	1/1	1/1
52F	0.213	0.998	447	5.2e + 13	1/1	1/1
04B	0.045	0.998	2742	7.1e + 09	1/1	1/1
36C	0.144	0.998	755	1.4e + 14	1/1	1/1
02A	0.289	0.998	1497	5.1e + 64	1/1	1/1
40B	0.237	0.998	988	2.7e + 33	1/1	1/1
37A	0.209	1.000	2900	1.4e + 84	1/1	1/1

Assays were performed to measure the fetal fraction, variant allele fraction, and number of input *HBB* DNA molecules. The likelihood ratio of sequencing error vs paternal inheritance was then used to infer the fetal genotype. The actual genotype of the fetus is confirmed by genotyping of the newborn.

NIPT for recessive inheritance of *HBB* was then performed on samples with the heterozygous rs713040 maternal genotype, T/C. In these samples, the fetal genotype could be (i) homozygous reference allele, T/T; (ii) heterozygous T/C; or, (iii) homozygous for variant allele C/C. To more closely match the clinical use case of determining fetal disease status for a recessive disorder, the T/T and T/C genotypes were considered ‘normal,’ whereas the C/C was considered ‘affected.’ As in paternal inheritance NIPT, recessive inheritance NIPT uses a statistical model to compute the likelihood ratio of an affected vs normal fetal genotype. Out of the 13 samples in which NIPT results and newborn follow-up were available, 5 were C/C affected, 3 were T/T normal, and 5 were T/C normal (Table 2, Fig. 6C–E). All NIPT results agreed with follow-up neonatal genotyping. Recessive inheritance NIPT analysis was performed on an additional 10 cfDNA samples, but the results were indeterminate based on the LR thresholds (Table S3). Notably, even lower amounts of cfDNA were recovered from the no-call samples (mean GE = 600) compared to the samples for which recessively inherited NIPT calls were made (mean GE = 1800). The strength of incorporating molecular counting into NIPT is that unreliable samples can be identified and incorrect calls can be avoided. Given that cfDNA obtained from *HBB* heterozygotes at Baylor College of Medicine had mean cfDNA molecular counts of 3500 GE (Fig. 5), we expect the no-call rate to not be an important issue in a clinical setting. Our mathematical analysis that uses the quantity of cfDNA found in the US data suggests that a clinical assay that combines carrier screening and single-gene NIPT in the US population, overall no-call rate would be less than 1% (Table S2). Overall, 27/27 NIPT calls, including paternal and recessive inheritance, were concordant with the newborn *HBB* genotype, suggesting that QCT analysis is highly accurate for sgNIPT even in challenging samples.

**Table 2:** NIPT for recessive inheritance of rs713040 from pregnant maternal blood.

Sample	Fetal Fraction	VAF	Num. Mol.	LR	NIPT	Neonate GT
30B	0.153	0.477	8616	1.0e − 44	normal	0/0
31B	0.171	0.478	304	1.5e − 02	normal	0/0
08A	0.134	0.478	2092	3.5e − 09	normal	0/0
56C	0.325	0.482	663	7.2e − 12	normal	0/1
39A	0.079	0.498	1868	2.3e − 02	normal	0/1
63B	0.225	0.505	1386	2.4e − 08	normal	0/1
47F	0.308	0.508	1319	6.2e − 14	normal	0/1
09B	0.208	0.521	1601	1.3e − 04	normal	0/1
38A	0.112	0.568	2328	7.9e + 07	affected	1/1
25B	0.242	0.569	360	1.7e + 01	affected	1/1
40E	0.184	0.570	573	2.1e + 02	affected	1/1
50C	0.179	0.586	1085	3.4e + 06	affected	1/1
17B	0.219	0.603	1611	1.8e + 14	affected	1/1

NIPT analysis was performed on pregnant mothers who are heterozygous for the rs713040 variant. NIPT was considered ‘affected’ if fetal genotype was inferred as homozygous variant (1/1), and ‘normal’ for heterozygous (0/1) or homozygous reference (0/0).

## Discussion

There is a significant unmet medical need for NIPT for sickle cell disease, beta-thalassemia, alpha-thalassemia, cystic fibrosis, and spinal muscular atrophy. Individuals affected by these disorders face significantly reduced life-span and require frequent access to intensive medical care. Current medical guidelines set by the American College of Obstetrician & Gynecologists recommend that all pregnancies should be screened to determine the carrier status of both mother and father before offering genetic counseling and invasive diagnostic procedures^16^. However, paternal carrier testing may be unavailable or unreliable. Paternity is not established at the time of birth in 30% of US births to single mothers^39^, and this is in addition to a significant risk of hidden non-paternity that has been uncovered in population-level genetic studies^40^. Moreover, reducing the need for paternal DNA could be a significant improvement in clinical practice. Current screens that test the mother for carrier status result in 11% to 30% positive calls and require follow-up with paternal carrier testing^41^.

Single-gene NIPTs require precision at the level of molecular counts. Digital PCR is an alternative method for obtaining molecular count data^26,27^. In fact, NIPT for sickle cell disease and beta-thalassemia has previously been demonstrated in proof-of-principle studies using dPCR^35,36,42^. However, dPCR has two critical limitations that hinder its translation to the clinic. First, dPCR relies on ~15–30 nt SNV-specific probes and therefore does not the have the single-base pair resolution of a sequencing approach. If nearby cis-variants are present within this footprint or if a high homology region is also amplified, a significant proportion of cases can be missed by dPCR. This problem is particularly acute in sickle cell disease, in which HbC, HbS, and rs713040 are all within 12 bp of each other; and the *HBD* gene is a close paralog of *HBB*. These factors might have contributed to the 80% accuracy obtained in a previous study that used dPCR for NIPT of sickle cell disease^42^. In addition, the multiplexability of digital PCR is limited to only 2–4 variants. This significantly limits its throughput and use for multiple variants, disorders, or samples at the same time, and has become a crucial impediment to its widespread clinical use.

To overcome these limitations, we developed a method for counting DNA molecules using amplicon-based next-generation DNA sequencing and QCT analysis. The QCT molecular counting approach uses synthetic DNA that co-amplifies with the gene-of-interest (e.g. *HBB*, *CFTR*, etc.) to serve as a reference standard. The exact number of QCT molecules added to each sample is reconstructed from sequencing data which then enables molecular count information of the gene-of-interest to also be recovered from sequencing depth data. Therefore, the synthetic QCT DNA used as a reference standard is calibration free and not susceptible to the Poisson noise associated with the addition of ~100–400 synthetic DNA molecules. Furthermore, because QCT molecular counting is compatible with amplicon sequencing, multiple loci can be simultaneously interrogated via multiplex PCR, and as many as 50–100 sgNIPT assays can be pooled and sequenced on a single Miseq lane (200,000–400,000 reads per assay). Single-gene NIPTs using QCTs were developed for sickle cell disease, cystic fibrosis, beta-thalassemia, alpha-thalassemia, and spinal muscular atrophy. *HBB* NIPT was performed on blood taken from pregnant women, and results were confirmed by follow-up DNA sequencing of newborns, resulting in 100% concordant NIPT calls. No paternal DNA was used for these NIPT calls. Molecular counting ability enables statistical modeling of NIPT results so that false calls can be avoided with even the most challenging samples.

In addition to improvements in single-base pair resolution and multiplexability, QCT molecular counting has 25–100x more dynamic range than digital PCR. Droplet digital PCR is most precise at measuring ~20,000 molecules, with an upper limit of 100,000 molecules^26^. Even low-throughput NGS instruments such as the Illumina MiSeq produce 25 million reads, which can be used to count 2.5 million molecules using the QCT method without any decrease in accuracy. This increase in dynamic range unlocks absolute quantification of DNA and RNA levels in a diverse range of applications including T-cell receptor profiling, transcriptomics, and microbiome applications.

Prior to QCT molecular counting, diverse adapter/primer pools called UMIs have been used for detection of unique molecules as well as for low resolution molecular counting^24,43^. QCTs rely on a similar computational approach in which sequence diversity is used to identify single molecules. However, the QCT approach embeds sequence diversity within the target region instead of requiring a ligation or pre-amplification step inherent to UMIs. This simpler QCT approach has several advantages, including (i) it avoids inefficient ligation and pre-amplification PCR steps that can result in >90% loss of the sample, resulting in lower sensitivity; and (ii) sequence diversity is contained within ~100 molecules in the QCT approach, as opposed to the picomole (10^11^ molecules) amounts of UMIs that can lead to primer/adapter dimers that hinder multiplexing^44,45^.

We also found that QCTs were useful for ensuring the integrity of sample preparation and sequencing in addition to molecular counting. These features of QCTs should enable more reliable and sensitive rare variant detection in liquid biopsy. Currently, the limit of detection (LOD) for circulating tumor DNA is typically given in terms of allele fraction. However, an LOD of 0.1% allele fraction is not meaningful when a particular sample only contains 500 haploid genomic equivalents (less than 1 molecule). The QCT molecular counting approach enables an LOD to be calculated as an absolute number of tumor DNA molecules. Furthermore, reliable rare-variant detection requires assurances that minor alleles are not the result of cross-contamination from previously processed or positive control samples. We have integrated QCT cross-contamination analysis into our workflow for detecting contamination from fluid handling, operator error, and index misassignment. Measurements of contamination and index misassignment in every sample, rather than only as a negative control, could enable a lower limit of detection to be obtained in liquid biopsy applications. Because QCTs are internal amplification controls that are present in every sample, they are also sensitive to and can help identify common problems that can degrade assay performance, such as PCR inhibition from hemolysis or salt and ethanol carryover during DNA purification.

Perhaps, the highest technical impact of QCT molecular counting may be on quantification of gene copy number variation (CNV). 5–10% of the human genome consists of CNV > 50 bp, and an additional 33% of the genome is susceptible to CNV in cancerous tissues^46,47^. Although CNVs are often seen as hallmarks of cancer, the capabilities of current liquid biopsy approaches for CNV detection are limited. Our NIPT results, particularly those that require copy number variation detection such as spinal muscular atrophy and alpha-thalassemia, suggest that QCT molecular counting can detect even a single additional copy of a gene at 5% allele fraction. This is a significant improvement over current liquid biopsies that can only detect 6 additional copies or more of an oncogene, e.g., *HER2*, at tumor fractions of 5–20%^14,15^.

## Methods

### QCT synthesis and analysis

QCT pools were synthesized by Integrated DNA Technologies as 4 nmol scale ultramers. Sequences for *HBB* Exon 1 QCTs are given in Supplementary Dataset 1. QCT oligos were double stranded by addition of primer hbs_qct_pext and incubation with Klenow polymerase at 37C for 1 hour. Double stranded QCT pools were then gel-purified and diluted to 15 fM in TE-Tween (10 mM Tris, 0.1 mM EDTA, 0.05% Tween-20).

QCT reads matching the QCT identifier were extracted from raw sequencing reads. For each PCR reaction, EMI sequence clusters were generated by grouping EMI sequences together if they differed by 2 or fewer mismatches. EMI sequence clusters were then thresholded into high- or low-depth clusters by read depth. The read depth threshold is computed for each PCR reaction as the square root of the mean EMI sequence cluster read depth. The number of QCT molecules in that PCR reaction is the number of high-depth EMI sequence clusters, and $\langle {D}_{QCT}\rangle$ is the mean read depth per QCT molecule. Contaminating QCT reads were identified by a low-read depth EMI sequence cluster in one PCR assay that appears in another PCR assay at high-read depth. The EMI fingerprint for the PCR reaction is the set of high read depth EMI sequences. The assayed genomic equivalents of *HBB* exon 1 DNA is computed as $\frac{{D}_{HBB}}{\langle {D}_{QCT}\rangle }$, where ${D}_{HBB}$ is the read depth of the *HBB* exon 1 amplicon.

### DNA Purification and Library Prep

Approximately 10 mL of venous blood was collected into a cfDNA blood collection tube (Streck, Omaha, NE). Plasma was separated from whole blood according to manufacturer instructions and stored at − 20C until further processing. Cell-free DNA was purified from plasma via the Qiagen Circulating Nucleic Acid kit using an elution volume of 50 ul.

An 86-plex amplicon NGS assay was designed to measure paternal inheritance of common allele frequency SNVs for fetal fraction measurements (Fig. S8). 10 ul of cfDNA was used per fetal fraction assay.

To perform the *HBB* exon 1 allele fraction assay, approximately 200 HBB QCT molecules were added to 35 ul of cfDNA, and the mixture was PCR-amplified and sequenced on the Illumina Miseq using dual unique indexes.

### Bioinformatic processing

FASTQ sequencing output is analyzed using a custom bioinformatic processing pipeline to measure pathogenic allele dosage, fetal fraction, molecular counts, and the presence of any paternally inherited alleles (Fig. 1). Briefly, sequencing reads are first filtered for QCT reads using the QCT identifier, and QCT analysis is performed to count the number of DNA molecules interrogated by the assay (see above). The remaining reads are then aligned to the human reference genome build GRCh37 using the Burrows Wheeler algorithm^48^. The allele dosage of the maternal variant is calculated as the fraction of variant reads over the total number of reads mapping to that region. A particular allele was classified as paternally inherited if the allele dosage was between 0.5% and 20%, as previously described^35^. Finally, the fetal fraction was determined as $2\hat{\varepsilon }$, where $\hat{\varepsilon }$ is the median MAF of paternally inherited alleles across 9 chosen loci^35^. These four parameters are then entered into an NIPT statistical model (see below).

### NIPT statistical modeling

Similar to previous digital PCR analyses of single-gene NIPT^35,36,42^, a likelihood ratio threshold was used to determine objective boundaries for negative, positive, and no-call results. Briefly, the binomial distribution was used to model the probability of measuring the *HBB* allele fraction, *x*; given fetal fraction, 2*ε*; and DNA molecule count, *N*. The likelihood of a paternal allele is then $$P(x|N,\varepsilon )=(\begin{array}{c}N\\ Nx\end{array}){\varepsilon }^{Nx}{(1-\varepsilon )}^{N\mathrm{(1}-x)}$$, and the probability of sequencing error is $$P(x|N,s)=(\begin{array}{c}N\\ Nx\end{array}){s}^{Nx}{(1-s)}^{N\mathrm{(1}-x)}$$. The rate of sequencing error, *s*, was set at 0.5%. The LR that the fetus inherited a paternal allele compared to sequencing error is the ratio of these two probabilities, $LR={(\frac{\varepsilon }{s})}^{Nx}{(\frac{1-\varepsilon }{1-s})}^{N\mathrm{(1}-x)}$. For recessive inheritance, we computed likelihoods for an affected vs a heterozygous fetus, $${p}_{aff}=(\begin{array}{c}N\\ Nx\end{array}){(\frac{1}{2}+\varepsilon )}^{Nx}{(\frac{1}{2}-\varepsilon )}^{N\mathrm{(1}-x)}$$ and $${p}_{het}=(\begin{array}{c}N\\ Nx\end{array})\,1/{2}^{N}$$. Samples that had likelihood ratio $1/8 < \frac{{p}_{aff}}{{p}_{het}} < 8$ were considered no-calls. Fetal fraction of *HBB* exon 1 was adjusted by a factor of 0.74 to match levels observed in pregnant samples without follow-up (Fig. S8).

### Non-pregnant cell-free DNA controls

Samples were obtained from 30 pediatric patients with SCD receiving care at Texas Children’s Hospital Hematology Center. Samples were collected from both genders, aged 2–18 years of age, and obtained under a Baylor College of Medicine Internal Review Board approved protocol. Informed consent was obtained from all participants and/or their legal guardian(s). All methods of the study were carried out in accordance with relevant guidelines and regulations. Venous blood was collected in 10 mL Streck cfDNA tubes, and cfDNA was purified from plasma using the Qiagen circulating nucleic acids kit. The HBB allele fraction assay was performed on all samples. The remaining cfDNA in these samples were either used in the fetal fraction assay as negative controls or analyzed by fluorometry (Qubit) to compare DNA mass and assayed genomic equivalents (Fig. 5B).

### Pregnant maternal blood samples

Buccal swabs and 6–10 mL of venous blood in Streck tubes were obtained from 208 pregnancies at >12 weeks of gestation at Yashoda Hospital, Ghaziabad, India. Samples were collected under a protocol approved by the Institutional Ethics Committee of Yashoda Hospital (IEC: ECR/970/Inst/UP/2017). Informed consent was obtained from all participants and/or their legal guardian(s). All methods of the study were carried out in accordance with relevant guidelines and regulations. Of these patients, we were able to obtain buccal swabs of 52 newborns for confirmation of NIPT results. *HBB* genotyping of the newborns was performed by PCR amplification of the *HBB* gene using primers HBBNextera500F1 and HBBNextera500R1 to generate a 2.5 kb amplicon, followed by Nextera-based library preparation and Miseq sequencing. The GATK Hapolotype Caller was then used to call variants from sequencing data^49^. Cell-free DNA was purified from maternal plasma by the Qiagen Circulating Nucleic Acids kit with an elution volume of 50 ul. 10 ul of cfDNA was used for measuring fetal fraction. 35 ul of cfDNA was used for the *HBB* exon 1 allele fraction assay.

## Supplementary information

Dataset 1

Supplementary Information
