# Supplementary material for: A novel high-throughput molecular counting method with single base-pair resolution enables accurate single-gene NIPT
Source: Sci Rep. 2019 Oct 7;9:14382. doi: 10.1038/s41598-019-50378-8 (PMC6779891; doi:10.1038/s41598-019-50378-8)
Supplement: Supplementary file 2 — Supplementary Information [file 41598_2019_50378_MOESM2_ESM.docx]

**Supplementary Information**


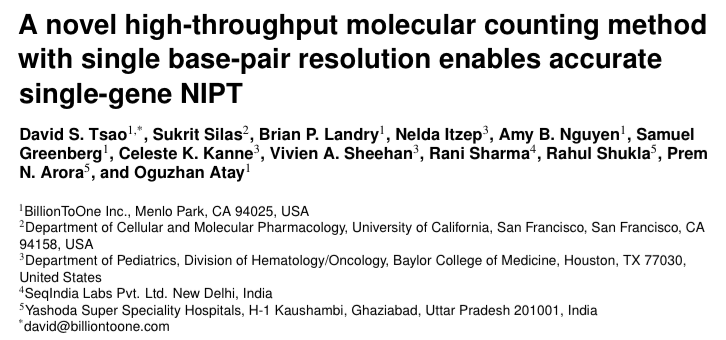


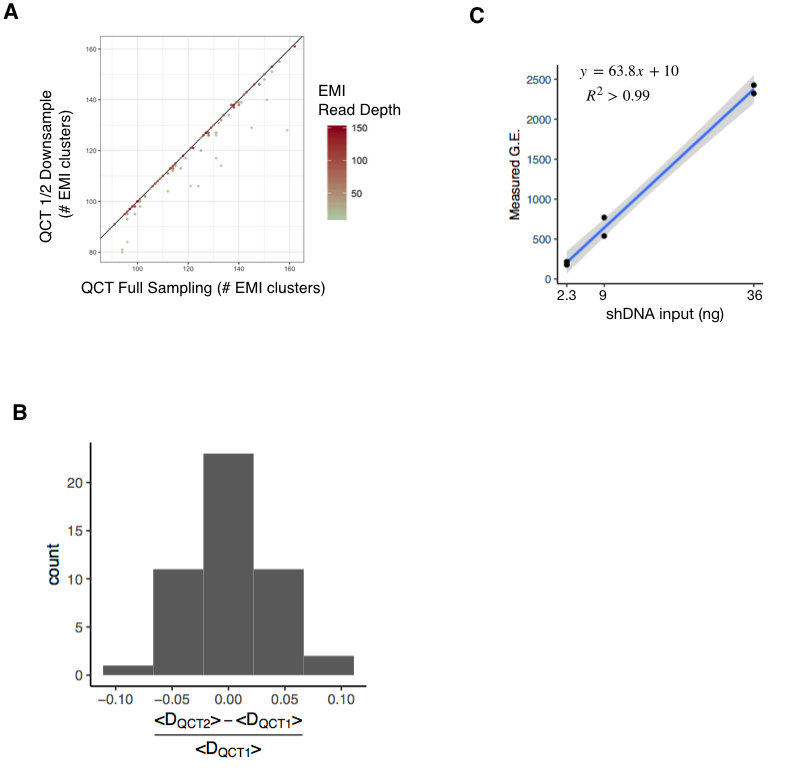


Figure S1: Molecular counting by QCTs. (A) Fastq sequencing reads from PCRs in Fig. 2 were downsampled to half the original read depth. The number QCT molecules in each PCR was determined by counting the high-depth EMI clusters for both the original and downsampled sequencing data. The black line has slope=1 and y-intercept=0. (B) QCT1 and QCT2 pools were included in PCRs, and the mean read depth per QCT pool, $<D_{QCT}>$, was measured for each PCR. The difference in mean read depth for QCT1 and QCT2 thus serves as an internally controlled estimate of error in $<D_{QCT}>$ measurements. The histogram shows the relative difference of $<D_{QCT1}>$ and $<D_{QCT2}>$ for 48 PCRs. The mean relative difference was 0.006%, with standard deviation of 3.7%. (C) Human genomic DNA was sheared using a Covaris instrument to ~150bp. A dilution series of 2-36ng human DNA was prepared, and the number of assayable genomic equivalents for those DNA masses was measured using HBB QCT analysis. About 64 GE/ng of HBB DNA was assayable in these PCRs.


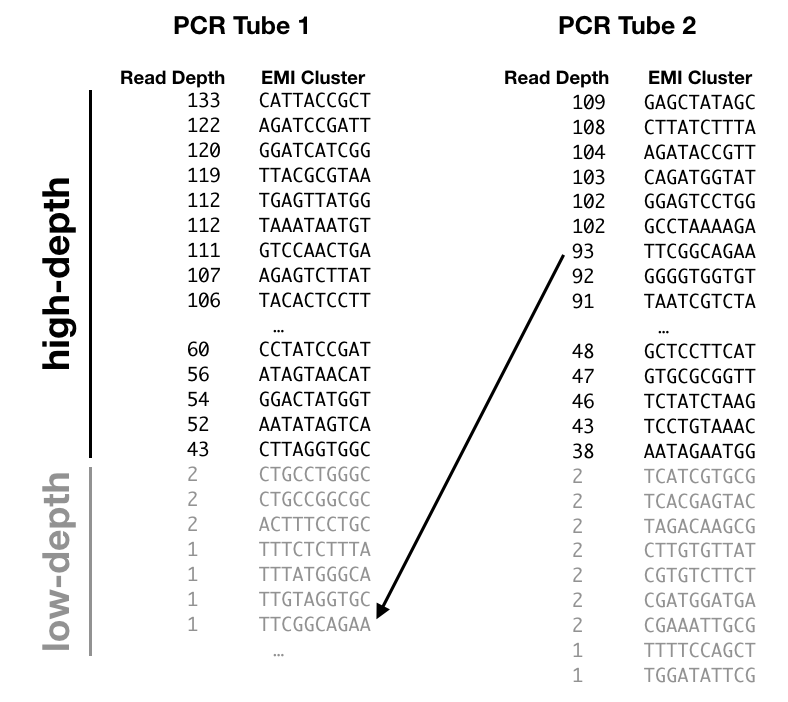


Figure S2: Example of EMI sequence cluster thresholding and contamination detection. For each PCR, the read depth of each EMI sequence is first determined. EMI sequence clusters are then formed by grouping together EMI sequences that differ by 2 or fewer mismatches. Finally, EMI sequence clusters are classified as high-depth or low-depth by a read depth threshold (see Methods). Contamination across PCRs is identified when a low-depth EMI cluster is observed at high-depth in a different PCR (arrow).


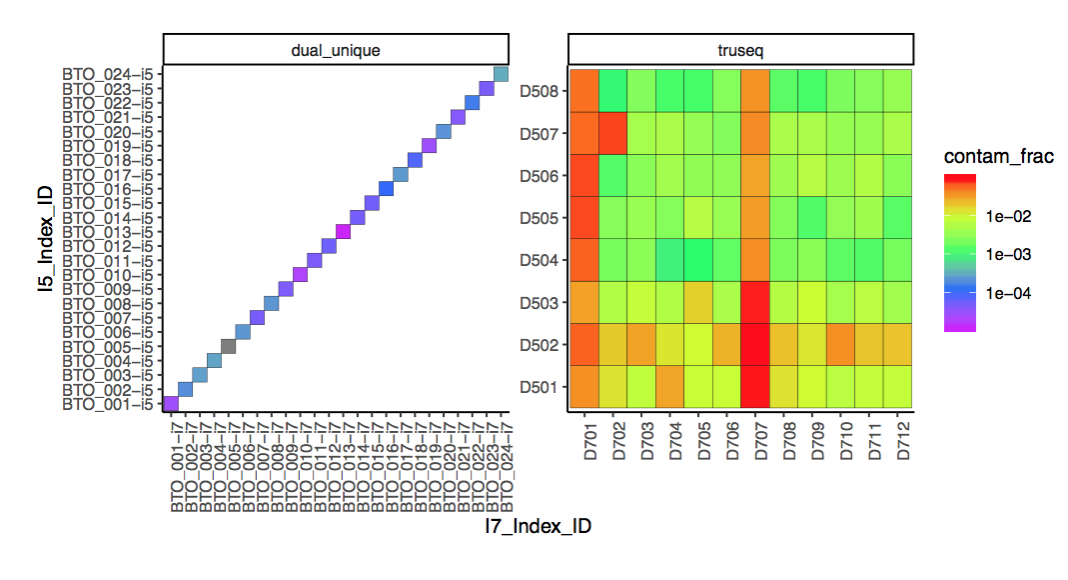


Figure S3: Comparison of observed contamination between combinatorial TruSeq and dual unique indexing. PCRs amplifying QCT molecules were prepared in a single batch and were indexed using either unique dual indexes or Truseq D7xx/D5xx combinations. The contamination fraction for each PCR was calculated as number of contaminating QCT reads over total QCT reads.


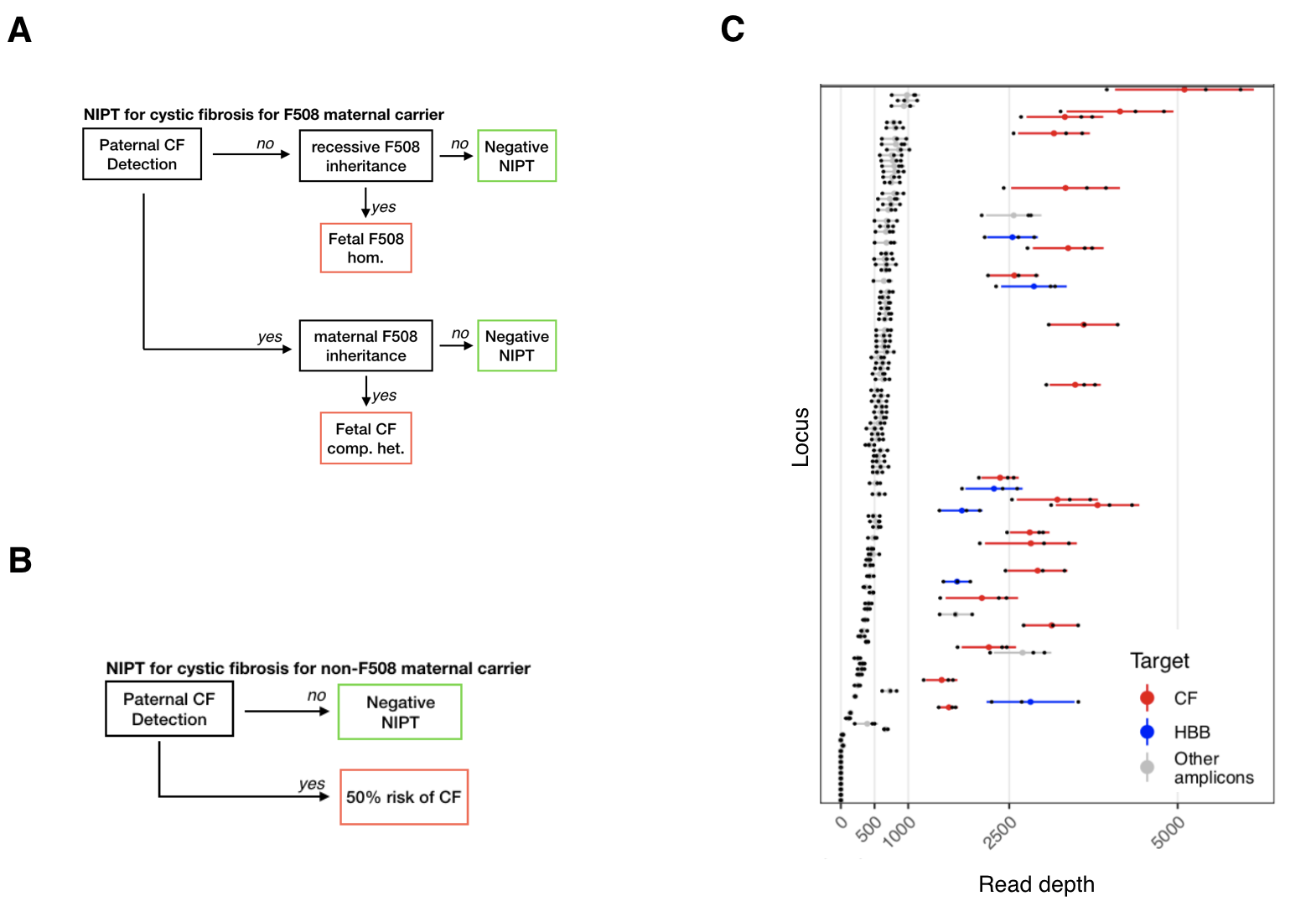


Figure S4: NIPT for cystic fibrosis. The majority of cystic fibrosis (CF) chromosomes are due to the F508del variant^1^. CF NIPT was therefore designed to detect homozygous F508del and compound heterozygotes for the most common CF variants. (A) Cystic fibrosis NIPT diagnostic algorithm for a maternal carrier of the F508del variant. Possible fetal genotypes in this case are (i) WT/WT, (ii) F508del/WT, (iii) WT/X, (iv) F508del/F508del, or (v) F508del/X; where X is a cystic fibrosis variant besides F508del. Affected fetuses can have genotypes (iv) or (v). The Paternal CF detection assay is a multiplex PCR that screens for the most common pathogenic variants responsible for cystic fibrosis. If a distinct, paternally inherited fetal allele for CF is detected, then maternal F508 inheritance analysis is used to determine whether the fetus has the WT/X or F508/X genotype. If no paternal pathogenic CF allele is detected, then the fetus can have genotypes i, ii, or iv. Recessive inheritance of F508del is then used to distinguish between WT/WT or F508del/WT and F508del/F508del genotypes. (B) Diagnostic algorithm for a maternal cystic fibrosis carrier besides F508del. If the maternal CF genotype is WT/Y, then the possible fetal genotypes are: (i) WT/WT, (ii) WT/X, (iii) Y/X, or (iv) Y/Y, where X is a paternally inherited CF variant allele and Y is the maternal CF variant allele. Affected genotypes are (iii) and (iv). Detection of paternal CF variant, X, can therefore result in WT/X or X/Y fetal genotype with equal probability. If inheritance of a paternal CF variant is excluded, then there is a small residual risk that the fetus could have inherited an identical non-F508del CF variant from both mother and father. (C) Paternally inherited CF alleles are detected using a multiplex PCR for simultaneous analysis of fetal fraction, CF, and HBB paternal alleles. The read depth of CF targets is >1000x, which is sufficient for detecting paternally inherited alleles at minor allele fractions >1%.


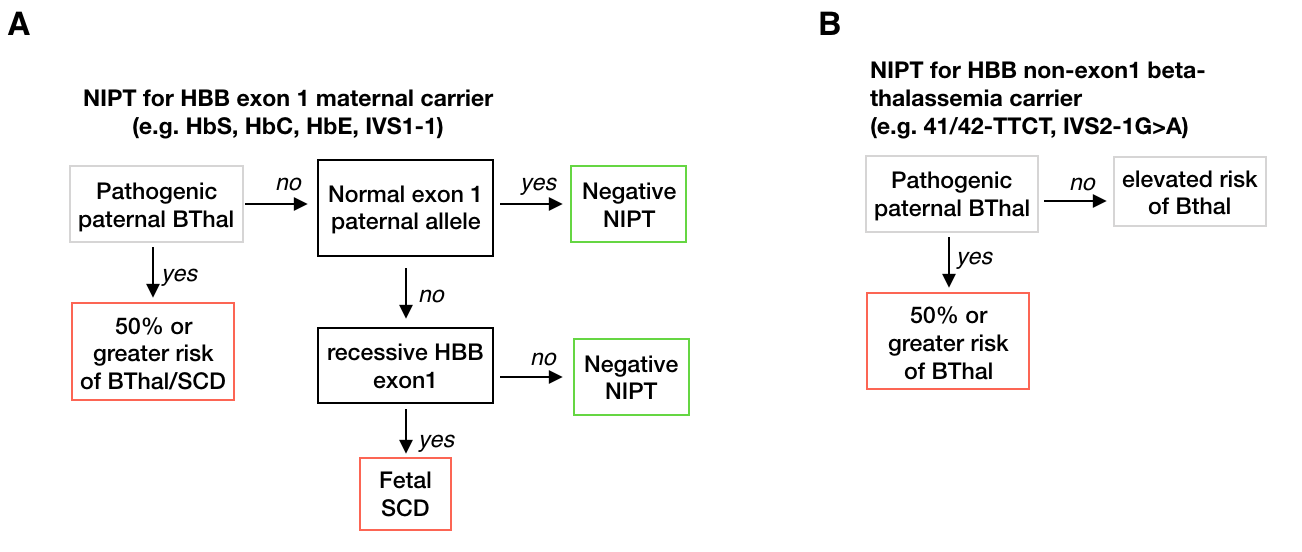


Figure S5: HBB NIPT for sickle cell disease and beta-thalassemia. Sickle cell disease is the most common hemoglobinopathy due to pathogenic variants in HBB. (A) NIPT diagnostic algorithm for a SCT mother. If the mother carries a pathogenic variant in exon 1 of HBB, NIPT proceeds by first excluding fetal inheritance of a pathogenic HBB paternal allele. If a pathogenic paternal allele is detected, a 50% or greater risk of BThal/SCD is reported out. If no pathogenic paternal allele is detected, the next step is to detect a normal exon 1 paternal allele (typically by paternal inheritance of the common rs713040 variant). If a normal, paternally inherited HBB exon 1 variant is detected, then the fetus cannot be affected and a NEGATIVE NIPT report is issued. Otherwise, a recessive HBB exon 1 analysis is performed to determine whether the fetus inherited two copies of the identical HBB variant from mother and father. (B) NIPT diagnostic algorithm for a Beta-thalassemia carrier. When the mother has a non-exon 1 pathogenic variant, HBB NIPT first excludes paternal inheritance of a different pathogenic allele. If a paternally inherited pathogenic allele is detected, then there is a ½ chance that the fetus also inherited the pathogenic maternal allele and is compound heterozygote for B-thal or B-thal/SCD. If no pathogenic paternal allele is detected, then an “elevated risk” of beta-thalassemia is reported, as it is still possible for the fetus to be homozygous affected. In such a case, confirming that the father is not a carrier or has a different variant from the mother would confirm that the fetus is unaffected.


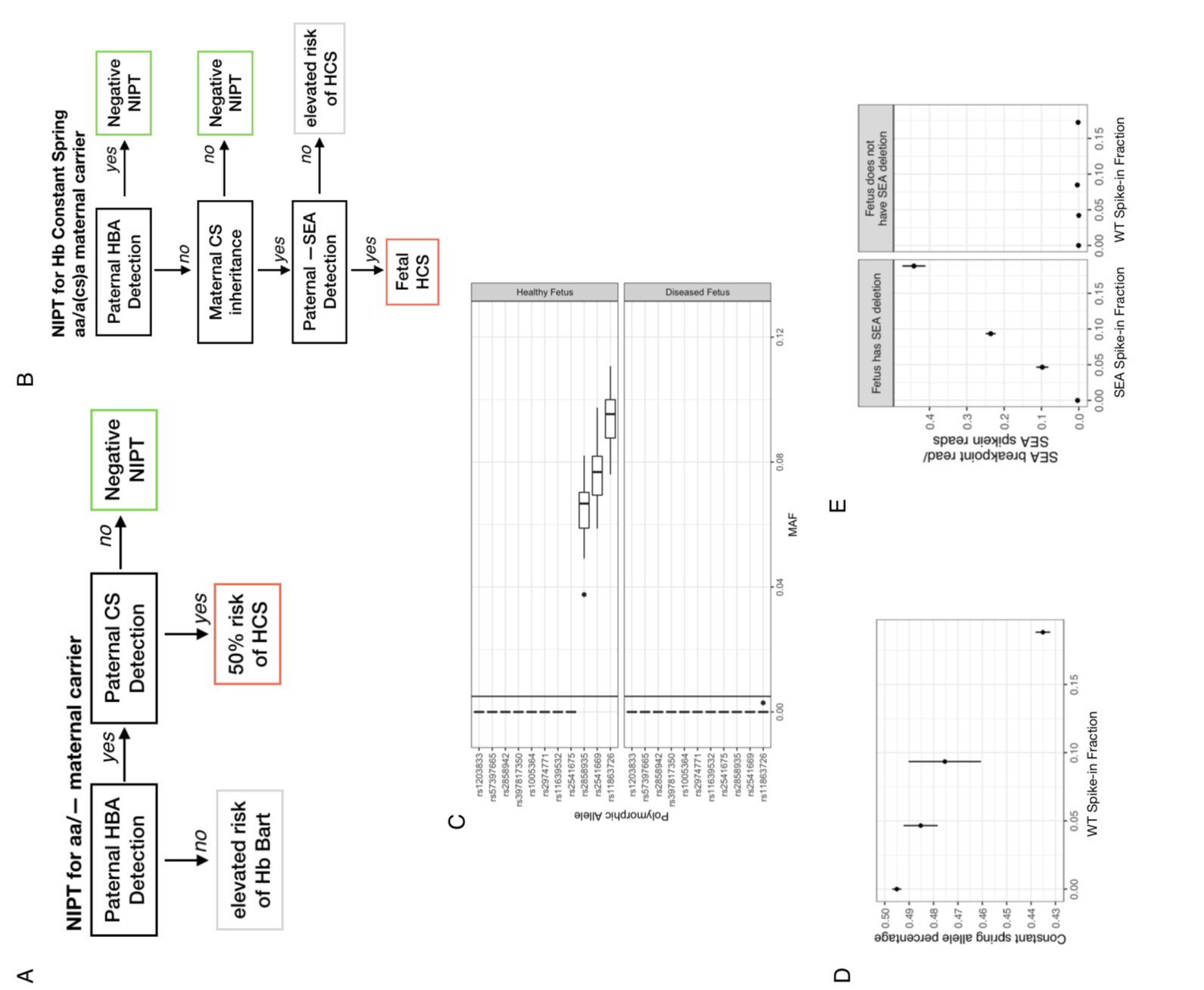


Figure S6: Design and validation of alpha-thalassemia NIPT. NIPT for alpha-thalassemia focuses on Hb Bart and HbH-Constant Spring (HCS), which are the most severe forms of the disease^2^. Diagnostic algorithms for fetal Hb Bart or HbH-Constant Spring for a double deletion maternal carrier (A) or a Hb Constant Spring maternal carrier (B). The alpha-thalassemia NIPT is comprised of three assays. When the mother has double deletion in cis, the alpha-thalassemia NIPT first uses the HBA paternal detection assay to determine whether 1 or more HBA copies were inherited from the father. If >1 HBA copy is paternally inherited, and no Hb Constant Spring allele is detected, then the fetus must have at least 1 HBA copy and is not at risk for Hb Bart. However, if a paternally inherited Hb Constant Spring is detected, then aa/a(cs)a or --/a(cs)a are equally likely. (B) If the mother is known to be a Hb Constant Spring carrier, i.e. genotype aa/a(cs)a, then the fetus may be at risk for HCS. The alpha-thalassemia NIPT first uses the HBA paternal detection assay to determine whether 1 or more HBA copies were inherited from the father. If >1 HBA copy is paternally inherited, then the fetus is not at risk for HCS. If a paternal allele is not detected and we determine the non-HCS maternal allele was inherited by the fetus, then the child is not at risk for HCS. However, if we determine the child inherited the HCS allele from the mother, and we also detect a SEA breakpoint deletion from the father, then the fetus has a high risk of having HCS. (C) Validation of paternal inheritance of HBA. There are 11 benign, common (AF > 0.2) SNVs that are spanned by the SEA deletion. If a paternally inherited SNV is detected in at least 1 of those SNVs, then the fetus must have inherited a non-double-deletional paternal allele. Sheared genomic DNA mixtures of aa/-- trait DNA (NA10799) was mixed with 10% aa/aa DNA (NA12878) or 10% --/-- DNA (NA10796 and subjected to the paternal inheritance assay. All three informative paternal alleles were detectable in the healthy fetus, and no informative paternal alleles were detected in the diseased fetus. (D) Constant spring maternal inheritance assay. Sheared genomic DNA corresponding to an aa/a(CS)a mother and a normal, aa/aa child were mixed at 5% to 20%. As expected, the allele fraction of the CS variant decreases as more wildtype DNA is added to the mixture. (E) Breakpoint PCR of –SEA. The –SEA is one of the most common deletions that span HBA1 and HBA2, and it’s breakpoint is well characterized^3^. Detection of fetal –SEA deletion can therefore be performed by PCR using primers that span the breakpoint. As an internal positive control, a SEA spike-in is also introduced that is amplified by the same primers. The assay successfully amplifies the breakpoint in DNA mixtures representative of 5% fetal fraction. No SEA breakpoint products were detected when –SEA DNA is absent.


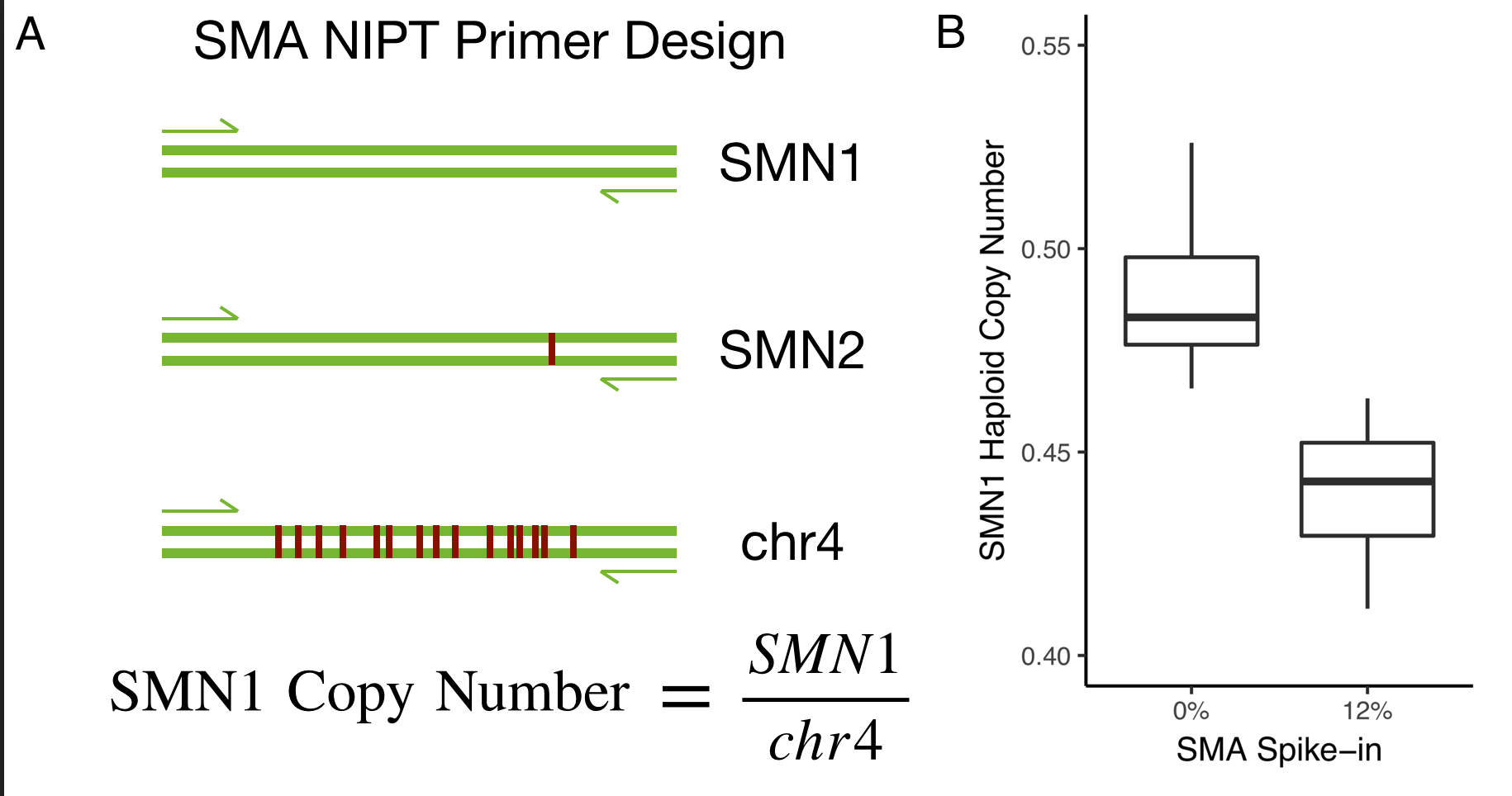


Figure S7: Fetal SMN1 measurement for Spinal Muscular Atrophy NIPT. SMA can be identified by homozygous deletion of SMN1^4^. A) The SMA NIPT primer pair co-amplifies SMN1, SMN2 and chromosome 4. SMN1 and SMN2 amplicons can be differentiated by the first variant in intron 7, chromosome 4 has identical priming sites and the amplicon has an edit distance of 15 from the SMN amplicons. The SMN1 copy number is calculated by looking at the ratio of the SMN1 reads to those from chromosome 4. SMN2 reads are not examined. B) Performance of the SMA NIPT on sonicated DNA samples mixed with a SMA carrier at 88% and a SMA individual at 12%. The decreased SMN1 copy number indicates that the “fetus” has the SMN1 0 copy genotype and therefore has SMA.


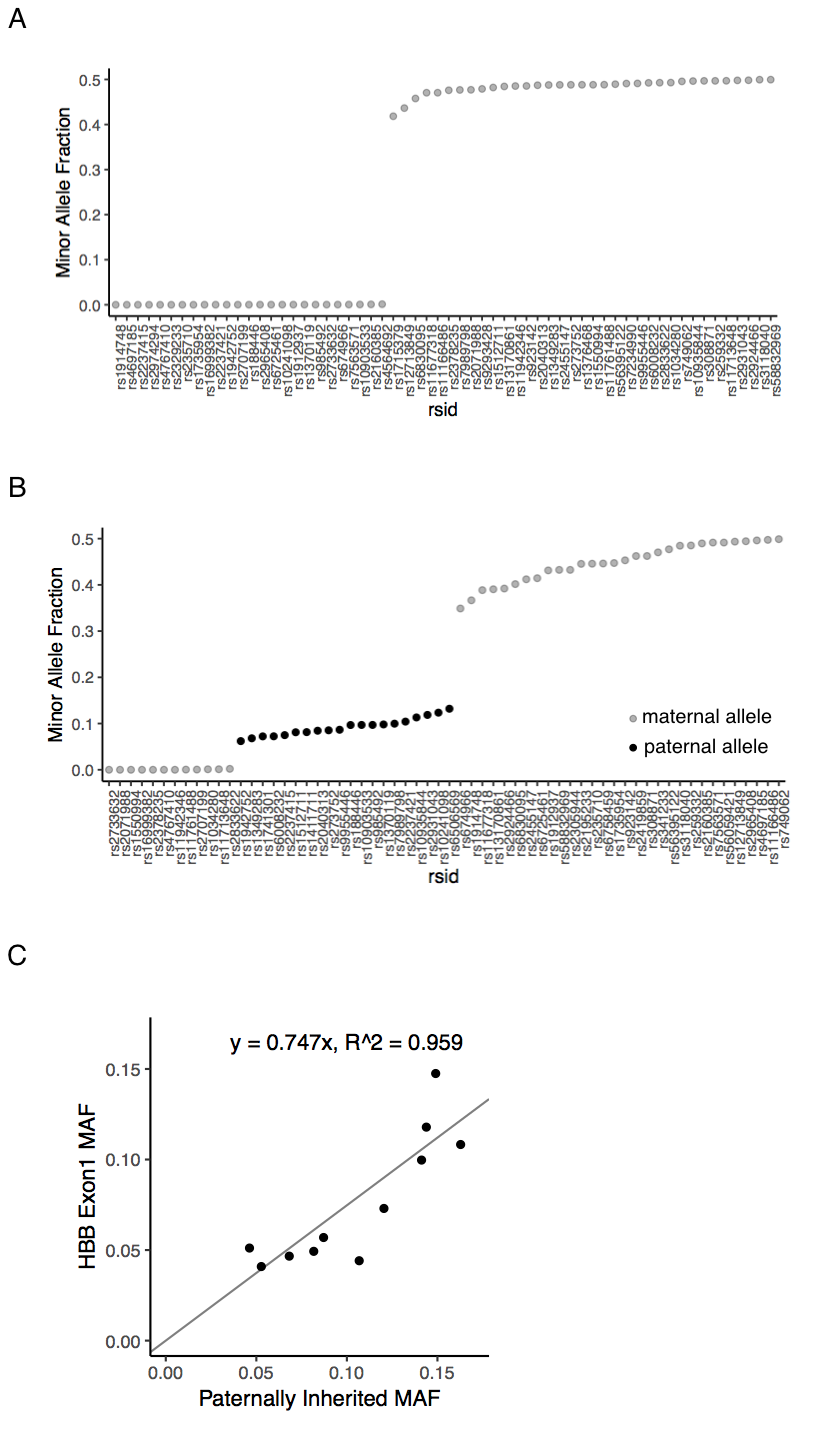


Figure S8: The fetal fraction assay interrogates >80 common frequency SNVs to detect paternally inherited SNVs, similar to what has previously been reported^5^. Paternally inherited fetal alleles are first identified as variants with 0.005 < MAF < 0.20, and the average allele fraction, $\hat{\epsilon}$ of paternally inherited alleles is estimated. The fetal fraction estimate is $2\hat{\epsilon}$. (A) The fetal fraction assay was performed on a non-pregnant cfDNA sample as a negative control. No paternally inherited alleles were identified. Only loci that pass filtering, i.e. read depth > 500, are plotted. (B) >20 paternally inherited fetal alleles were observed for a pregnany cfDNA sample. (C) Pregnant cfDNA samples for which no follow-up was obtained were still analyzed for HBB NIPT. 11 of these samples had distinct, paternally inherited fetal alleles in HBB exon 1 that enabled comparison of fetal HBB allele fraction with the genome-wide fetal fraction results.


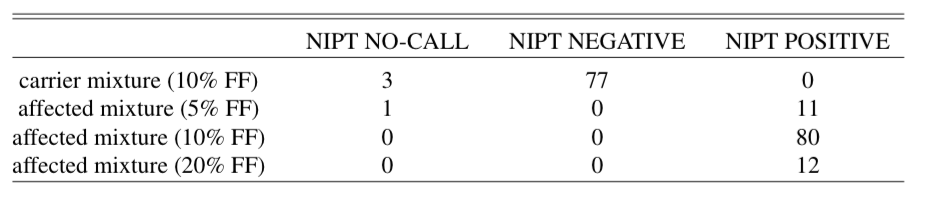


Table S1: NIPT calls for allele fraction measurements performed in Fig. 4D. The 0% HbS spike-in condition was taken to be an unaffected, carrier fetus at 10% fetal fraction. ‘NIPT Negative’ is returned when NIPT analysis determines that the fetus is either normal or carrier. ‘NIPT Positive’ is returned when NIPT analysis determines that the fetus is affected. NIPT analysis was 100% accurate with the prepared DNA mixtures, with only 4 no-calls out of 184 samples.


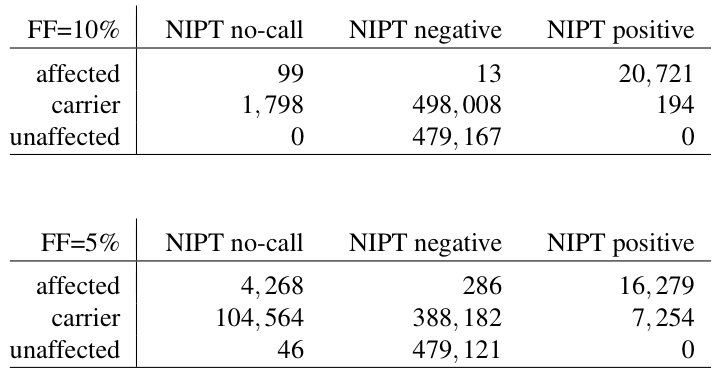


Table S2: Monte Carlo simulation of analytical sensitivity and specificity for recessively inherited NIPT from maternal blood alone. Expected numbers of affected, carrier, or unaffected fetuses were generated for 1,000,000 carrier pregnancies assuming a paternal carrier rate of 1/12. NIPT allele fractions were monte carlo simulated assuming 3500GE of cfDNA. The fetal fraction in simulations was set at either 10% or 5% to exemplify typical and most challenging scenarios. No-calls were determined by 1/8 $< LR< 8$. Sensitivity was >99% at 10% fetal fraction and >98% at 5% fetal fraction. Specificity was >99% even at the 5% fetal fraction condition. When the overall sensitivity and specificity of combined maternal carrier screening and NIPT is considered, the false positive rate should decrease by 1/12 (same as the carrier rate); therefore, a combined screen + NIPT workflow should have a specificity of >99.9%.


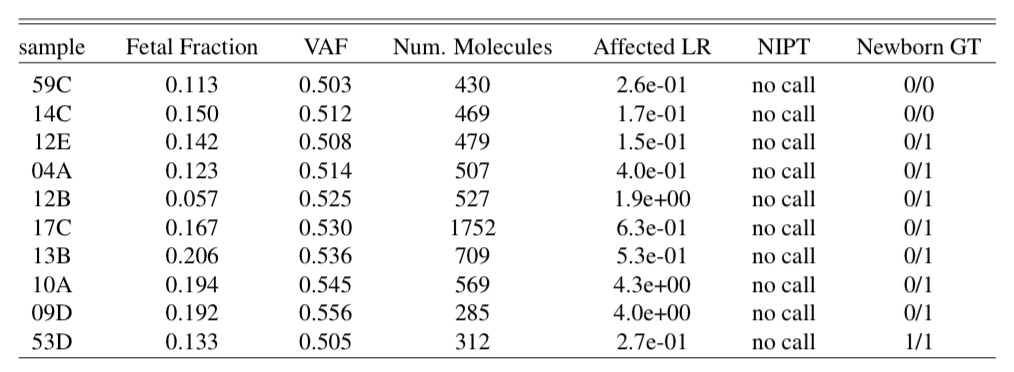


Table S3: Recessively inherited NIPT of HBB alleles from pregnant blood with 1/8 < LR < 8 that were considered ‘no-calls.’

1. Strom, C. M. *et al.* Cystic fibrosis testing 8 years on: Lessons learned from carrier screening and sequencing analysis. *Genet. Med.* **13,** 166–172 (2011).

2. Lal, A. *et al.* Heterogeneity of Hemoglobin H Disease in Childhood. *N. Engl. J. Med.* **364,** 710–718 (2011).

3. Chong, S. S., Boehm, C. D., Higgs, D. R. & Cutting, G. R. Single-tube multiplex-PCR screen for common deletional determinants of alpha-thalassemia. *Blood* **95,** 360–362 (2000).

4. Prior, T. W. Carrier screening for spinal muscular atrophy. *Genet. Medicine* **10**, 840–842 (2008).

5. Camunas-Soler, J. *et al.* Noninvasive prenatal diagnosis of single-gene disorders by use of droplet digital PCR. *Clin. Chem.* **64,** 336–345 (2018).
